# High Rates of Gene Flow by Pollen and Seed in Oak Populations across Europe

**DOI:** 10.1371/journal.pone.0085130

**Published:** 2014-01-13

**Authors:** Sophie Gerber, Joël Chadœuf, Felix Gugerli, Martin Lascoux, Joukje Buiteveld, Joan Cottrell, Aikaterini Dounavi, Silvia Fineschi, Laura L. Forrest, Johan Fogelqvist, Pablo G. Goicoechea, Jan Svejgaard Jensen, Daniela Salvini, Giovanni G. Vendramin, Antoine Kremer

**Affiliations:** 1 BIOGECO, UMR1202, INRA, Cestas, France; 2 BIOGECO, UMR1202, University of Bordeaux, Talence, France; 3 UR 1052, INRA, Montfavet, France; 4 Biodiversity and Conservation Biology, WSL Swiss Federal Research Institute, Birmensdorf, Switzerland; 5 Department of Ecology and Genetics, EBC, Science for Life Laboratory, Uppsala University, Uppsala, Sweden; 6 Alterra, Wageningen UR, Wageningen, The Netherlands; 7 Forest Research, Northern Research Station, Roslin, Midlothian, Scotland, United Kingdom; 8 Institute for Plant Protection, CNR, Sesto Fiorentino (Firenze), Italy; 9 NEIKER-Tecnalia, Vitoria-Gasteiz, Spain; 10 Forest & Landscape, University of Copenhagen, Copenhagen, Denmark; 11 Institute of Biosciences and Bioresources, CNR, Sesto Fiorentino (Firenze), Italy; The Ohio State University, United States of America

## Abstract

Gene flow is a key factor in the evolution of species, influencing effective population size, hybridisation and local adaptation. We analysed local gene flow in eight stands of white oak (mostly *Quercus petraea* and *Q. robur,* but also *Q. pubescens* and *Q. faginea*) distributed across Europe.

Adult trees within a given area in each stand were exhaustively sampled (range [239, 754], mean 423), mapped, and acorns were collected ([17,147], 51) from several mother trees ([3], [47], 23). Seedlings ([65,387], 178) were harvested and geo-referenced in six of the eight stands. Genetic information was obtained from screening distinct molecular markers spread across the genome, genotyping each tree, acorn or seedling. All samples were thus genotyped at 5–8 nuclear microsatellite loci. Fathers*/*parents were assigned to acorns and seedlings using likelihood methods. Mating success of male and female parents, pollen and seed dispersal curves, and also hybridisation rates were estimated in each stand and compared on a continental scale.

On average, the percentage of the wind-borne pollen from outside the stand was 60%, with large variation among stands (21–88%). Mean seed immigration into the stand was 40%, a high value for oaks that are generally considered to have limited seed dispersal. However, this estimate varied greatly among stands (20–66%). Gene flow was mostly intraspecific, with large variation, as some trees and stands showed particularly high rates of hybridisation.

Our results show that mating success was unevenly distributed among trees. The high levels of gene flow suggest that geographically remote oak stands are unlikely to be genetically isolated, questioning the static definition of gene reserves and seed stands.

## Introduction

Plants are static organisms whereas their genes are often highly mobile. Gene flow predominantly occurs through dispersal of both seed and pollen, and the contemporary distribution of neutral genetic diversity across the landscape is largely, though not entirely, due to the extent and relative importance of these two dispersal processes [1]. At the species level, seed and pollen immigration play crucial evolutionary roles, as the former is responsible for the colonisation of new habitats, whereas the latter may contribute to subsequent immigration, hybridisation and introgression. Mating systems and local patterns of gene flow are also important as they affect levels of inbreeding, family structures and local effective population sizes, which in turn impact local adaptation [2]. The genus *Quercus* dominates European forests, both for surfaces and for wood production income, and its importance is expected to increase with climate change [3]. Gene flow is an important factor to be taken into account for sustainable management of forest tree populations. Detailed studies of local gene flow performed in both pure and mixed stands of European white oaks indicate that pollen immigration into stands is generally high but very variable between studies [4]–[9]. Groups of fathers pollinating mother trees are significantly different from one mother to the other, but pollen clouds from inside and outside the study plot are not significantly differentiated [4]. Seed flow by gravity (barochorous) as well as jays and rodents (ectozoocory) is expected to be much lower than wind-related pollen immigration in oaks. Differences in acorn weight between species might explain the disparity in seed displacement measured between oak species [5]. Finally, hybridisation in mixed stands is asymmetric, variable and particularly dependent on both the relative abundance and the intimacy of the mixture of the congeneric species [10]. Our aim was to measure the extent of gene flow in white oaks (*Quercus robur* (pedunculate oak, 73.5% of the total tree sample), *Q. petraea* (sessile oak, 23%), *Q. pubescens* (pubescent oak, 2%), *Q. faginea* (Portuguese oak, 1.5%)) in eight stands distributed throughout the species' natural ranges across Europe, using the same molecular markers to perform paternity and parentage analyses. Stands with more than one species of oak are not particularly common across Europe, which constrained the choice of woods available for this study. Ideally, stands of similar size and density containing equal numbers of trees belonging to the same two species of oaks in intimate mixtures would have been chosen, but such stands rarely occur. Consequently, the best stands available to us were selected for inclusion in this study. We expected to control the heterogeneity across different ecological, demographic and sampling settings to enable general conclusions to be drawn.

We aimed to use our data to search for systematic trends in rates and patterns of gene flow in oaks. In addition, we introduce a novel approach, based on simulations, to draw confidence limits around dispersal curves and to determine whether observed values lie within these limits.

## Material and Methods

### Studied stands

**Figure 1:**
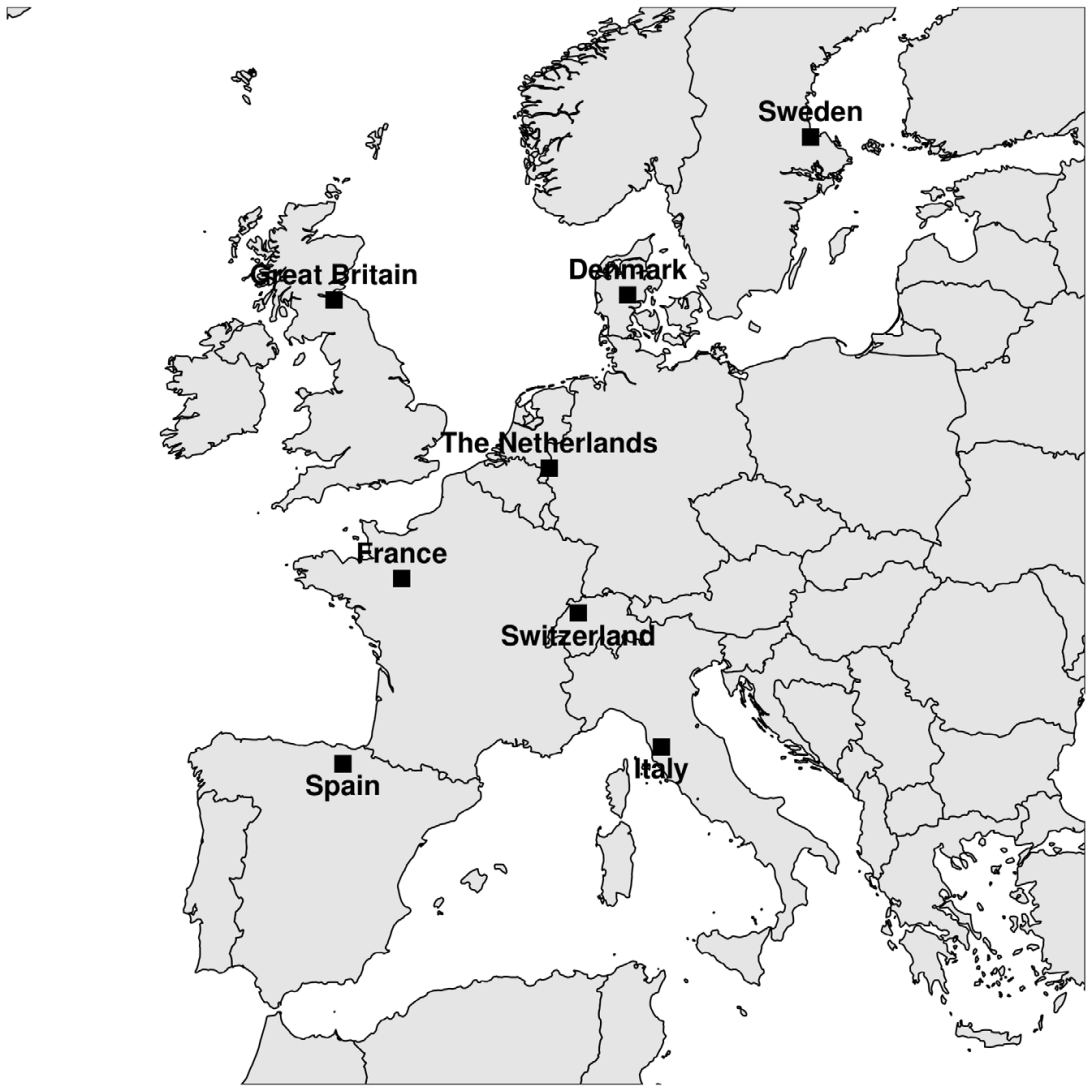
Location of the eight studied white oak stands across Europe.

Oak stands were selected in eight European countries (Denmark, France, Great Britain, Italy, The Netherlands, Spain, Sweden, Switzerland; Fig. 1 and Table 1) on the basis of the following common criteria: stands of natural origin should comprise *Q. petraea* and *Q. robur* (or respective congeners) in approximately equal proportions, adult trees should have a minimum age of ∼100 years, and sample size for each species should be close to 200, with all trees being analysed within a defined experimental area.

**Table 1:** Description of oak stands.

Country	Stand name	Size of the studied area (ha)	Number of *Q. petraea* trees	Number of *Q. robur* trees	Trees of intermediate morphology	Tree density (trees/ha)
France	Petite Charnie	5	166	183	5	70.8
Italy	Tatti	6	184	70**	41	50.8
The Netherlands	Meinweg 1998/2002	4.5	182	187	-	82
Spain	Gasteiz	∼50*	50***	189	-	-
Great Britain	Dalkeith	23	50	704	-	32.8
Denmark	Velling	5	120	245	-	73.0
Sweden	Båtfors	10	-	631	-	63.1
Switzerland	Bueren	9	76	349	17	49.1

5 *Q. robur* islands (11.24 ha, 1 ha, 4 ha, 4.4 ha, trees along a 500m road),

1 *Q. faginea* island (26.8 ha).

Q. pubescens.

Q. faginea.

The different stands included in the present study were subjected to tree labelling and tissue sampling for DNA analysis.

Land owner status and permission to work in the stands are the following:

- In Denmark, Velling is a state forest managed by the Danish Nature Agency (*Naturstyrelsen*), who gave the permission to conduct the experiment;

- in France, Petite Charnie is a state forest, the national forest agency (*Office National des Forêts*) gave the permission to conduct the experiment;

- in Great Britain, Dalkeith Oakwood is on private land owned by the Duke of Buccleuch. He gave written permission to work in the wood.;

- in Italy, we had a permission to work in the Tatti forest given by *Comunità Montana Alta Val di Cecina*;

- in The Netherlands, Meinweg is part of a national park, the permission to work was given by the state forest service (*Staatsbosbeheer*);

- in Spain, the forest of Gasteiz is a public area owned by the city council of Vitoria-Gasteiz, no particular permission was needed;

- in Sweden, experiments in Båtfors forest were performed thanks to a permission given by *Upplandsstiftelsen*;

- in Switzerland, Bueren is a public ownership forest, the forest service (*Forstdienst, Burgergemeinde Büren an der Aare*) gave its consent to the work;

The *Quercus* species we studied are neither endangered nor protected and no other protected or endangered species was concerned by our study.

It should be noted that despite our best efforts, it was not possible to find woods in each of the eight countries that fully met all the above criteria. The stands ranged in size from 5 to 50 ha. *Quercus petraea* and *Q. robur* were preferred, but alternative *Quercus* species were selected (*Q. pubescens*, *Q. faginea*) if only one of the two main species was present. Taxonomic identification of trees was based on leaf morphology and multivariate analyses [11]–[13]. All adult trees within the study area were labelled and mapped.

### Plant material

Ripe acorns were sampled from adult mother trees in each stand for paternity analysis. The number of mother trees varied between three and 44, depending on fecundity of trees during the sampling years and on trees' accessibility (Supporting Information, Fig. S1). The total number of acorns collected per stand ranged from 264 to 1543 (Supporting Information, Table S1 and S2 in File S1). Seedlings up to 20 cm high were mapped and sampled for parentage analysis in a subset of the study stands (France, Italy, Spain, Great Britain, Denmark, Sweden). The spatial distribution of the seedlings sampled was constrained by the existence and distribution of natural regeneration. Consequently, in some cases they were regularly sampled across a grid system whereas in other stands this was not possible as the seedlings occurred in scarce patches (Supporting Information, Fig. S2 and Table S1, Table S2 in File S1). DNA was extracted from leaves of all adult trees in the stands, also from leaves of sampled seedlings and from cotyledons of embryos in the seed samples. Collection of material was done between 1994 and 2003, depending on stand and on type of material. In one stand (The Netherlands, Meinweg), acorn collections were performed on two occasions four years apart (Supporting Information, Table S2 in File S1).

### Microsatellite genotyping

Unlinked, variable microsatellite markers were used to provide individual DNA profiles for adult trees, acorns and seedlings. The laboratory in the country of origin of the stand was responsible for the genotyping of the samples from that stand and selected loci that worked best under their laboratory conditions, using between 5 to all of the panel of 8 loci: MSQ4, MSQ13 [14], ssrQpZAG104, ssrQpZAG9, ssrQpZAG1/5, ssrQpZAG36 [15], and ssrQrZAG20, ssrQrZAG11, ssrQrZAG30, ssrQrZAG39, ssrQrZAG96 [16]. The selected microsatellites are robust and allele calling was standardized, but this was only necessary across individual microsatellite data sets within (and not across) individual stands, because allelic data were only used for paternity and parentage analyses within individual stands. Genotypic data were never compared between different stands and therefore uniformity in allele calling across laboratories was not an issue. Electrophoresis and silver staining or automated sequencer analyses are described in [17] and [18]. All loci are unlinked [19] and did not deviate from random mating according to Hardy-Weinberg equilibrium (data not shown).

### Data analysis

To estimate contemporary gene flow in the studied stands, data analysis was performed in two steps. The first step comprised parentage analysis, with either father assignment to seeds collected on known mother trees, or parents assigned to seedlings, with no means of distinguishing which of each parent pair acted as the mother and father. This step allowed us to estimate overall gene flow and hybridisation in mixed stands. The second step consisted of estimating pollen and seed dispersal kernels using the results of the parentage analysis. For this purpose, the highest probability to get the observed parentages was deduced from a mass-action model [20], and the parameters of the dispersal kernels were estimated by maximum likelihood. Possible family structure among adult trees was not taken into account in the analyses.

### Paternity and parentage analyses

**Paternity analysis.** Paternity analyses followed a standard procedure applied to each stand. We used the method of [21], implemented in the software FaMoz [22]. The efficiency of the markers for accurate identification of paternity and parentage in each stand was estimated beforehand by calculating exclusion and identity probabilities. We determined thresholds of acceptance with or without mistyping, percentage of pollen immigration, percentages of fathers correctly identified (*Correct father choice, Cfc*) and correctly assigned paternity among the assigned paternities (*Paternity correctly assigned, Pca,* when fathers assigned to offspring are truly their father among the cases where fathers were assigned to simulated offspring).

**Determining thresholds.** In a first analysis, simulated offspring (100,000 for each simulation) were generated using firstly parent genotypes and secondly population allele frequencies for each stand. We assumed either that there was no mistyping or that mistyping occurred at a rate of 0.001%, an arbitrary value that is convenient to avoid exclusion, due for instance to a single incorrect mismatch, and is a sufficiently small value to limit noise in calculations and hence false assignments. Simulations were used to build a statistical test, defining a lod-score threshold (likelihood ratio) and a threshold for delta (difference between lod-scores of the most likely father and the second most likely father, see [23]) to assign fathers to offspring within each stand, i.e. among the genotyped parents.

The estimation of gene flow from outside the stand, i.e. the percentage of offspring with no father assigned, was deduced from father assignments using three tests, one based on lod-scores, the second on delta-scores, and the third on the combination of both lod-scores and delta-scores. In case of a tie between potential fathers for a given offspring, no father was assigned.

**Paternity test simulation.** For each stand, simulations were run to mimic the experimental design (number of mothers, number of offspring per mother), i.e. use the gene flow estimation to create offspring with a father from the genotyped parents or with a father from "outside" the stand. The percentages of correct father choice (*Cfc*) and the percentages of correctly assigned paternity among the assigned paternities (*Pca*, number of genotyped fathers assigned to offspring and being truly their father compared to the total number of times genotyped fathers were assigned to simulated offspring according to the test) were calculated with or without mistyping and with the three types of tests (lod-score, delta-score or both); the test giving the highest percentages was used in the final paternity analysis. We further computed the *β* error (cryptic gene flow or false assignment, i.e. assignment of a genotyped father when the true father is outside the stand) and the *α* error (false rejection, i.e. assignment of no father when the true father is inside the stand).

**Parentage analysis.** As for paternity analysis, simulations (two sets of 100,000 simulated offspring with either genotyped parents or genotypes created using allele frequencies) were used to determine test thresholds for assigning a single parent or a parent pair to a seedling, again using FaMoz [21], [22].

Both lod-scores (likelihood ratio) and delta-scores (difference between lod-scores of the most likely parent and the second most likely parent) were used to assign parents. Tests mimicking the true data were built to evaluate the quality of assignments compared to true relationships, using single-parent and parent-pair lod-scores, delta-scores or both, with a null mistyping rate or with a low error rate of 0.001%. The rate of correct parent/parent pair choice (*Cpc and Cppc* respectively) corresponding to parent/parent pair correctly assigned either inside or outside the studied stand was evaluated. The tests providing the highest correct assignment rates and the smallest type I and II errors (*α*, *β*) were selected for the final analyses in each stand.

**Gene flow estimation after parent assignments.** After the parentage test, each seedling was assigned either to no, one or two genotyped parents. When a single parent was assigned, and because no sufficiently polymorphic uniparentally inherited markers were available, we assumed that this represented the maternal contribution on the basis that acorns are large, heavy seeds, oaks are wind-pollinated and the seedling is therefore likely to grow closer to its mother than to its father [24]. This assumption introduces a possible bias towards overestimation of pollen vs. seed immigration. Total gene flow, more specifically seen here as gene immigration, was split into seed and pollen contributions: seed flow was inferred from the percentage of seedlings with no parents assigned within the stand, and pollen immigration was calculated as the percentage of seedlings with either no parent or a single parent assigned within the stand. Total gene immigration can then be seen as a combination of seed and pollen immigration.

### Dispersal curve analyses

Dispersal analyses were performed at the stand level. To model pollen and seed dispersal, we calculated the probability of a pollen grain (or seed) from a given tree to reach a given position. Probabilities were thus estimated conditional on the position of trees present inside the stands, and the data came from two sources: seeds collected from trees with a father assigned and seedlings with two parents assigned.

**Pollen dispersal likelihood for paternity data.** Each tree *i*, located at *O_i_*, releases a pollen cloud and we assume that:

(i) each pollen grain, originating at *O_i_*, has a probability density *h_θ_*(*x*-*O_i_*) to reach a point *x*, where *h_θ_* is a probability density function known up to a given set of parameters *θ_,_*, and there is no effect of other trees on dispersal,

(ii) all trees produce the same quantity of pollen,

(iii) a pollen grain has a probability *q* of fertilizing a given flower from another species (hybridisation),

(iv) a pollen grain of a given mother tree will fertilize a flower from the same tree (selfing) with a probability *s* (*s*  =  0 if it is another tree from the same species).

Mother tree *j*, located at *x* = *O_j_*, receives from trees located at *O_i_* a global effective pollen cloud, which is the sum over *i* (tree *i*) of the following expression:



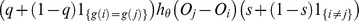



with *g(j)* being the genotype of the mother.

We consider only the effective pollen cloud that can produce offspring, with a pollen fraction *s* from the mother tree itself and a pollen fraction *q* from the other species [25].

A male tree *i* will fertilize a female tree *j* with a probability conditional on its contribution to the total amount of fertile pollen, that is:



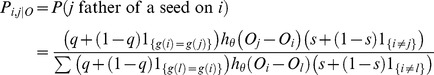



In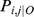 the term '|*O*' indicates that the fertilization model depends on the positions of all trees when the flowers are pollinated by fathers from the stand.

If we assume that fertilizations are independent between flowers, the probability *P*_1_ of observing, for all seeds *g*, on trees *M*(*g*), the fathers *P*(*g*) among trees *O_i_* is simply:



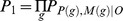



Briefly, we made the following assumptions: trees produce equal amounts of pollen and fecundities are equal among trees (estimation of fecundity was beyond the scope of this project and therefore no correlated pollen production covariate — such as tree height for instance — was available for inclusion in our models); sampling of pollen grains and seeds is random and all events are independent. Even though it is known that paternal and maternal reproductive output varies among trees [26], we refrained from deliberately introducing such unknown bias. Only seedlings with two assigned parents present inside the stand (and thus including the mother) were used for the calculation of seed dispersal.

**Dispersal distributions and dispersal functions.** When a father is assigned to a seed, the pollen dispersal distance equals the distance between the mother tree (on which the seed was collected) and the assigned father. Thus, paternity analyses provide direct measures of pollen dispersal distances caught by the sample. In parentage analysis, there is an ambiguity attached to father and mother assignments, pollen and seed dispersal were thus estimated assuming that of the two identified parents the mother was closer to the seedling than the father.

**Seed and pollen dispersal likelihood for parentage data.** Every mother *j* located at *O_j_* disperses her acorns in such a way that a seed reaches position *x* from mother *j* with a probability density of 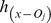. Fecundities are assumed to be equal among trees (same mean number of pollen grains or seeds for all trees).

A potential seed density 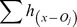 is associated to every point *x*. The seed that will give rise to a seedling is randomly selected among all the potential seeds. It originates from mother *j* with a probability proportional to the contribution of this mother to the seed rain. Even if fecundities are supposed to be equal, in a particular point *x*, the contribution of a given mother will depend on its distance to *x*. Therefore:



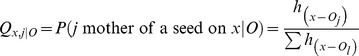



Only seedlings with two parents inside the stand (and thus including the mother) are used for calculations. The sum over *l* refers to the trees of the stand [27].

The probability for a seedling *u* to have a mother *M*(*u*) and a father *F*(*u*) can be written as: 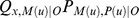. If *P*1(*u*) and *P*2(*u*) are the parents of a seedling, each one can be the father or the mother. The probability of observing these two parents is therefore:



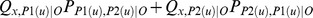



Conditional on the fact that the possible parents of the seeds are known, the probabilities that the seeds have the set of parents *P*1(*u*) and *P*2(*u*) are assumed to be independent. Therefore the probability for each observed seedling *u* to have the couple of observed parents (*P1*(*u*), *P2*(*u*))_u_ can be written:



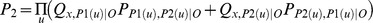



We fitted the dispersal data to a one-dimensional power exponential function:



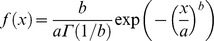



where *x* is the pollen or seed dispersal distance, *Γ*() is the gamma function, and *a* and *b* are the distance and the shape parameter, respectively. If *b* = 1, *f*(*x*) is an exponential function, if *b* = 2, *f*(*x*) is a Gaussian function and if *b*<1, the function is fat-tailed [28].

The mean distance *δ* travelled by pollen or seed is expressed by:



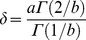



Distance *a* and shape parameter *b* were estimated using a maximum likelihood method, and the mean distance travelled by pollen or seed (*δ*) was deduced.

**Dispersal parameter estimation.** The parameters estimated by maximizing the likelihood of the complete data set (paternity and parentage likelihood) were the percentage of selfing, *s*, the percentage of interspecific hybridisation, *q*, and the distance and shape parameters of the dispersal distribution, *a* and *b*.

Confidence intervals of parameters were obtained for each stand by 1000 independent bootstraps [29]. For each stand, every simulation of pollen and seed dispersal was performed using the actual numbers of trees, seeds sampled per tree and seedlings sampled within the stand, using dispersal kernels with estimated parameters.

**Validation.** For each stand, a set of observed pollen dispersal distances was available for which a non-parametric density function was computed. The set of density functions of pollen dispersal distances obtained from simulations was used to compute a 95% individual confidence limit under the model assumptions. Comparisons between observed statistics and their expected distribution under the model were made by checking whether observed values were located within confidence limits [30]. We used the distribution of distances between mothers and fathers (respectively mothers and seedlings) calculated from the seeds collected from mothers (respectively from seedlings having two parents assigned) as a statistic to validate pollen dispersal (respectively seed dispersal). The mother was assumed to be the closer of the two parents assigned to the seedling. Pollen dispersal corresponds to the distance between the two identified parent trees. The distributions of pollen dispersal statistics were obtained by simulations using the dispersal model parameters estimated above. For each simulation, a father was assigned to seeds collected from mother trees and a father/mother pair was assigned to seedlings. Pollen and seed dispersal distributions were computed for each simulation. Validation was based on consistency between observed and model-predicted distributions.

## Results

### Paternity and parentage analysis

**Quality of assignment tests.** Exclusion probabilities for single parent, paternity, and parent pair were high, reaching almost 100%, while identity probabilities were very low in all eight study stands (Supporting Information, Table S3 in File S1). These values indicate that our genetic markers are informative, with high average capability to exclude any given paternity or parentage relationship [31].

For each stand and paternity assignment, we used the most successful test in terms of percentages of correct father choice (*Cfc*) and of father correctly assigned among the assigned paternities (*Pca*). Tests with no mistyping and using either fathers' delta-scores or both delta- and lod-scores for assignment proved to be the most reliable (Supporting Information, Table S4 in File S1). Mean *Cfc* was 88.3% with a standard deviation (SD) of 9.8 and mean *Pca* was 86.6% (SD  =  12.2). The quality of the tests varied among stands, but in most cases correct choices were > 80%. *α* (false assignment) and *β* (cryptic gene flow, *Cgf*) errors were less than 5% except in two cases for each parameter (Italy and Great Britain for *α*, Sweden and Switzerland for *β*). Observed gene immigration, i.e. the percentage of pollen grains from outside the studied plot or the percentage of acorns with no assigned father among the genotyped parents, was deduced from the decisions made with the tests applied to the different data sets.

For four out of six stands analysed for seedlings, the most reliable parentage tests were those based on lod-scores with no mistyping according to simulations, whereas two cases involved tests with a non-null mistyping (Supporting Information, Table S5 in File S1). The stand in which the highest number of loci were analysed (Denmark, eight loci) provided the most accurate estimations of these rates. The *α* and *β* (*Cgf*) errors associated with these tests were 15.5% and 6.2% on average, with large variation depending on the stand analysed, especially for *α* (0.5–35% for *α* versus 0–13.1% for *β*).

The utilised markers have low levels of null alleles as inferred from *FIS* values and associated non-significant heterozygote deficiency tests (data not shown). A high incidence of null alleles can lead to an overestimation of *FST* and therefore to an underestimation of gene flow [32].

**Gene flow.** Mean pollen immigration deduced from paternity analysis was 57% over all stands (SD  =  20) i.e. on average, no father could be assigned to more than half the sampled acorns (*pi*, Table 2). Pollen immigration varied greatly among stands, ranging from 21% in Denmark to 81% in Spain. The differences in stand configurations do not explain this variation: mean pollen immigration was not significantly correlated with stand size. Stands analysed with five loci had a mean pollen immigration significantly smaller than stands analysed with six loci (54% compared to 75%), suggesting an effect of the number of loci on gene flow estimation. But inconsistently the Danish stand, analysed with eight loci, exhibited the lowest level of pollen immigration among all stands.

**Table 2:** Results of paternity analyses.

Site	*Nof*	*pi (%*)	*Nf*	*Nf/Ngp* (%)	*Nof*/*Nf* (SD)	*Ne*
France	328 (33%)	66.9	120	33.9	2.7 (3.4)	47.0
Italy	511 (61%)	39.2	159	53.9	3.2 (4.7)	51.6
The Netherlands 98	211 (30%)	69.9	92	24.9	2.3 (2.8)	37.7
The Netherlands 02	155 (35%)	64.8	68	18.4	2.3 (2.6)	30.1
Spain	50 (19%)	81.1	21	8.8	2.4 (2.7)	9.3
Great Britain	671 (59%)	40.9	286	37.9	2.4 (2.7)	125.0
Denmark	438 (79%)	20.9	178	48.8	2.5 (2.5)	89.4
Sweden	238 (22%)	77.6	158	25.0	1.5 (1.1)	101.5
Switzerland	723 (47%)	53.1	175	40.3	4.1 (7.1)	44.7
Mean		57.2	139.7	32.4		59.6
SD		20.1	76.4	14.5		37.5

**Figure 2:**
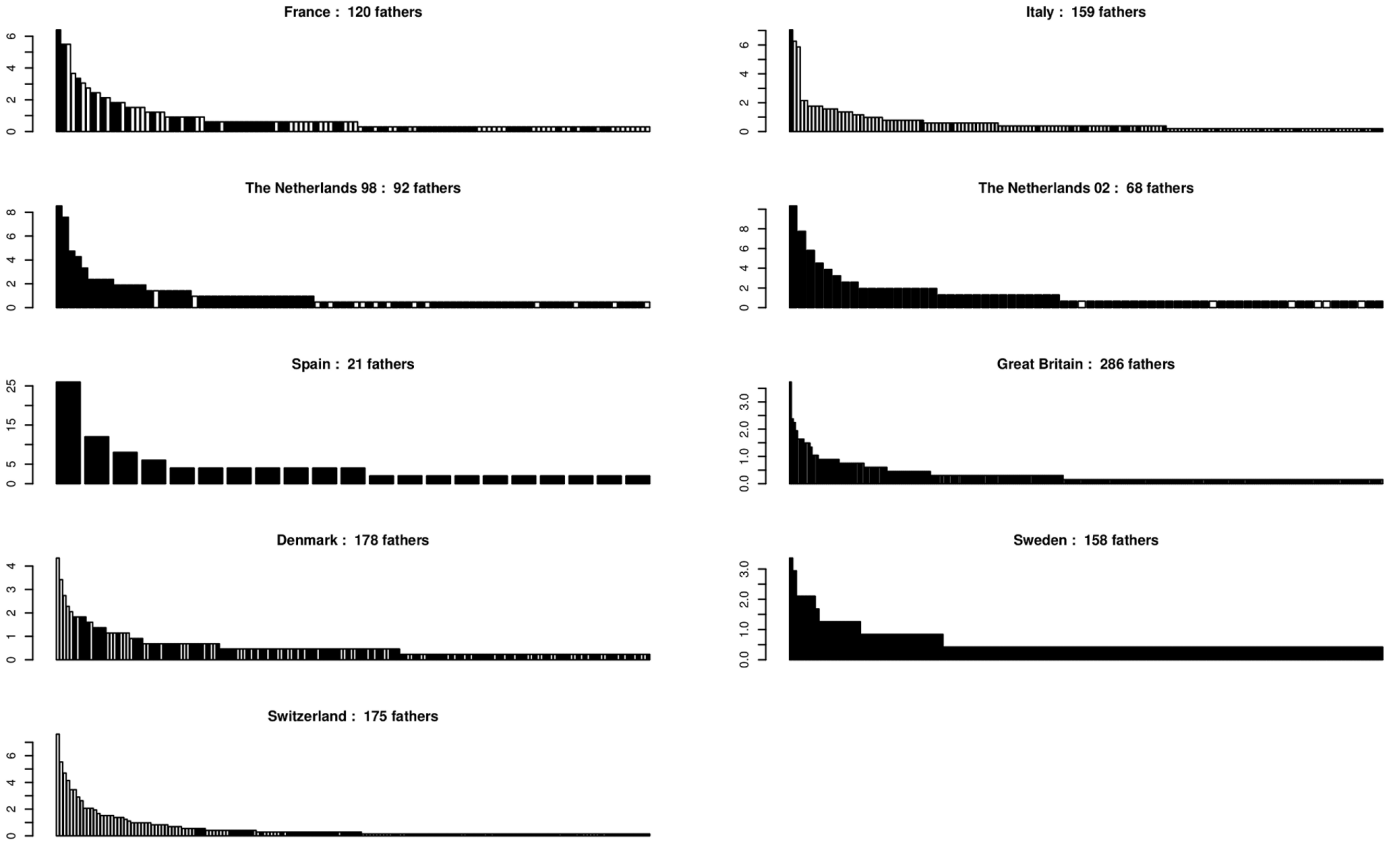
Variation of the relative reproductive success of male parents. The number of different fathers assigned after paternity analysis is given for each stand. In each stand, each bar is a father, fathers are ordered by decreasing reproductive success, bar height represents the percentage of the total number of acorns sired by the father. □ *Quercus petraea* ▪ *Quercus robur* 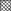 Undetermined species.

*Nof*: number of offspring with a father assigned (%). *pi*: pollen immigration. *Nf*: number of different fathers assigned. *Ngp*: Number of genotyped parents. *Nf*/*Ngp* (%). *Nof*/*Nf*: mean number of offspring per father (standard deviation (SD)). (see Fig. 2: male reproductive success). *Ne*: effective number of fathers (1/Σ(*f_i_* ^2^), where *f_i_* is the relative reproductive success of each father).

Mean gene immigration, deduced from parentage analysis, reached 61% (SD  =  15), ranging from 44% in Italy to 81% in Spain (Table 3). Seed and pollen immigration rates were deduced from the percentages of seedlings with no, one or two parents assigned (Table 3). Over all seedlings analysed, 46% on average (from 29% in Spain to 59% in Great Britain) were assigned to a single parent, and only 15% (from 2% in Denmark to 33% in Italy) had both parents assigned, leaving a mean of 39% of seedlings with no parent assigned. Thus, while mean seed immigration deduced from these assignments was 38% (from 20% in Italy to 66% in Spain), pollen immigration estimates in the seedlings were very high with an average of 85% (ranging from 67% in Italy to almost 98% in Denmark). Hence, a great discrepancy existed between mean pollen immigration estimated from paternity analyses of acorns (57%) and parentage analysis (85%). The assumptions underlying the analyses associated with the high heterogeneity of experimental parameters in our large-scale study could in part explain such a discrepancy.

**Table 3:** Results of seedlings parentage analyses.

		Assignments (numbers (%))						
Site	*Ns*	One parent	Two parents	*s* %	*h* %	Seed immigration (%)	Pollen immigration (%)	Total gene flow (%)
France	165	94 (57%)	15 (9%)	6.7	6.7	33.9	90.9	62.4
Italy	387	183 (47%)	126 (33%)	9.5	4.8	20.2	67.4	43.8
Spain	65	19 (29%)	3 (5%)	100*	-	66.2	95.4	80.8
Great Britain	191	112 (59%)	51 (27%)	5.9	47.1	14.7	73.3	44.0
Denmark	84	42 (50%)	2 (2%)	0.0	0.0	47.6	97.6	72.6
Sweden	175	63 (36%)	30 (17%)	6.7	-	46.9	82.9	64.9
Mean		46%	15%	21.5	14.6	38.2	84.6	61.4
SD		16	9	38.6	21.8	19.2	12.3	15.0

*Ns*: number of seedlings. *s*: percentage of selfing. *h*: percentage of hybrids, SD: standard deviation.

The three seedlings with two parents assigned were the result of selfing

In parentage analyses, selfing estimated on small sample sizes was highly variable and thus not reliable (21.5%, SD  =  38.6). In paternity analyses, as expected, few selfed acorns were produced with only 2.5% (SD  =  1.4) of crosses with a father assigned identified as being the same tree as the mother.

**Figure 3:**
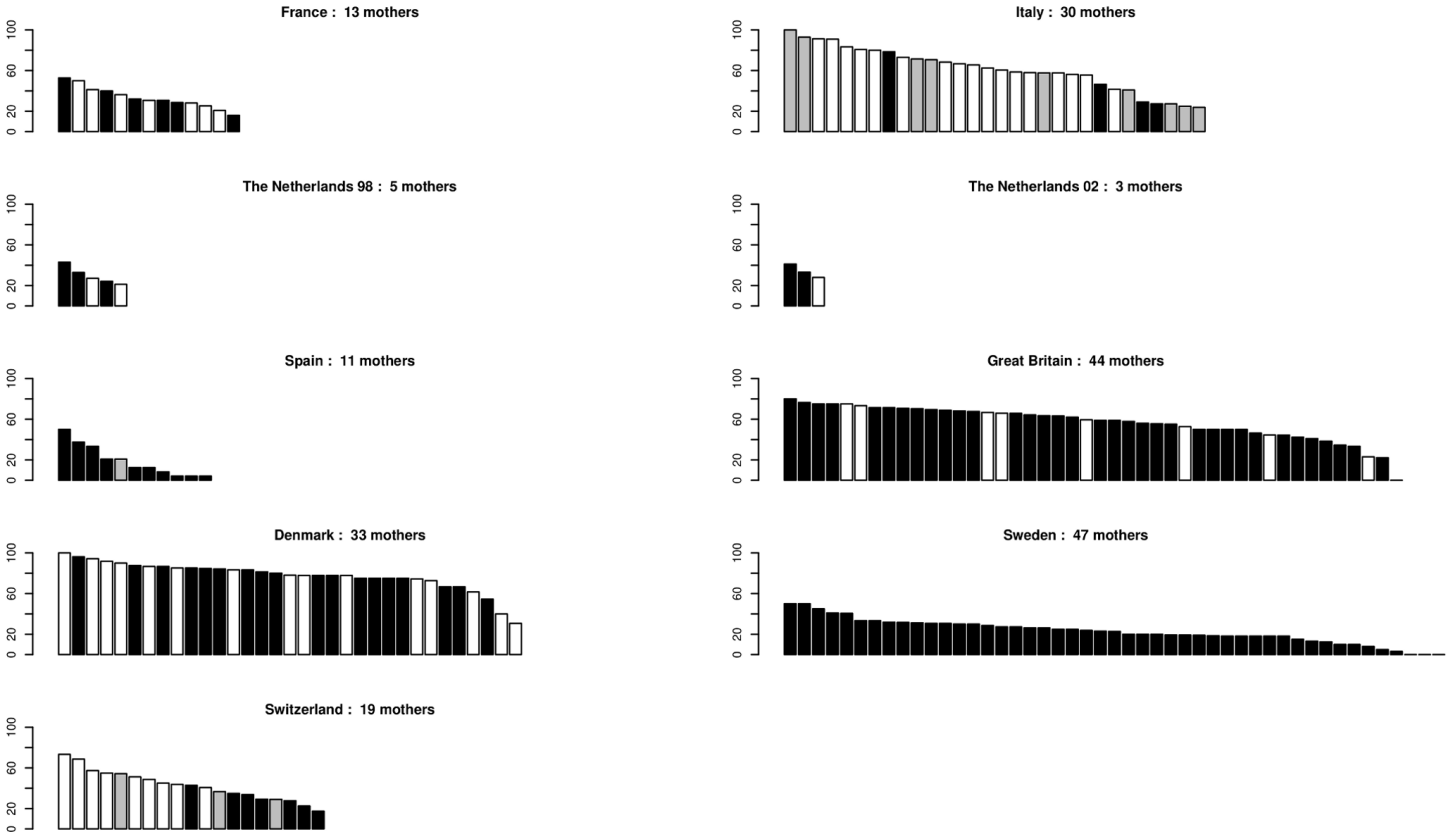
Variation of the percentage of fathers assigned per mother tree. In each stand, each bar is a mother tree, bar height represents the percentage of acorns collected from the mother tree with a father assigned. □ *Quercus petraea* ▪ *Quercus robur* 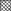 Undetermined species.

**Variation of mating success.** On average, 32% of genotyped trees contributed to reproduction although this estimate varied greatly among stands (from 9% in Spain to 54% in Italy, SD  =  15, *Nf*/*Ngp*, Table 2). There was a positive correlation (*r*  =  0.77, *p*  =  0.02) between the number of genotyped trees (*Ngp*) or the number of mother trees (*Nm*, Supporting Information, Table S2 in File S1) and the number of different fathers assigned (*Nf*, Table 2). This means that those stands which contained a higher number of sampled trees or mother trees tended to have a larger range of fathers contributing to the acorn or seedling crop. The reproductive success of these fathers (number of offspring sired) revealed L-shaped distributions in all situations (Fig. 2), with few fathers siring several offspring and the majority of fathers producing few offspring on the sampled mothers. In all stands, many of the fathers sired only a single offspring. The effective number of fathers (1/Σ(*f_i_* ^2^), where *f_i_* is the relative reproductive success of each father) was hence reduced to 43% of the number of different fathers siring the sampled mothers, but again with notable variation among stands. However, the locations of mother trees were not always uniform across each stand and therefore these figures are likely to be underestimates of the true effective number of fathers. The mean number of offspring per father (*No*/*Nf*, Table 2) was very stable among stands despite large variation in sample sizes. In Italy and Denmark, the percentages of acorns with assigned fathers reached almost 100% in some mother trees but assigned fathers could be as low as 20% for other mother trees in the same stands. Differences were also observed in the other stands, but they tended to be smaller (Fig. 3).

**Figure 4:**
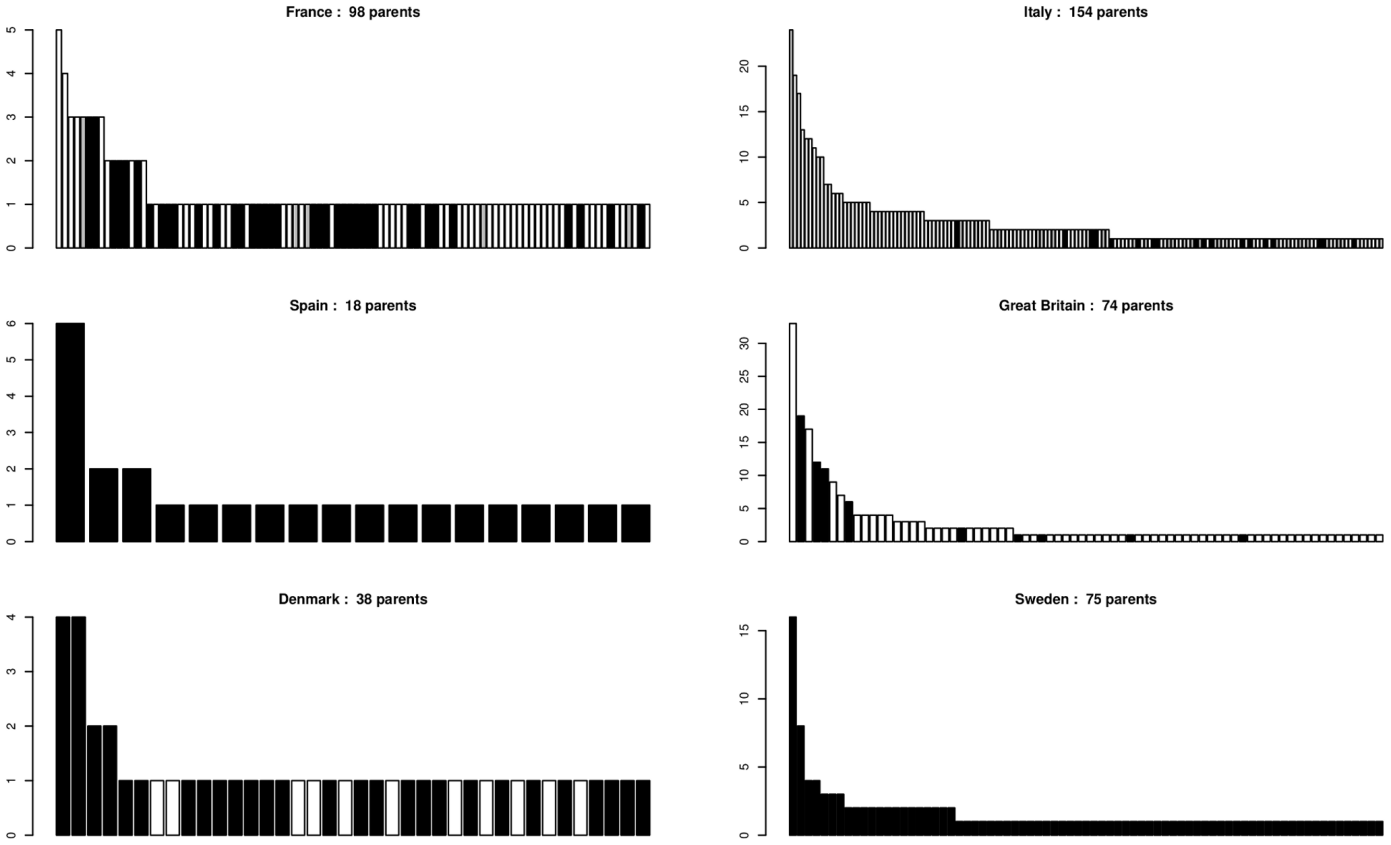
Variation of the relative reproductive success of trees. The number of different parents assigned after parentage analysis is given for each stand. In each stand, each bar is a parent, parents are ordered by decreasing reproductive success, bar height represents the percentage of the total number of seedling for which the considered individual is the parent. □ *Quercus petraea* ▪ *Quercus robur* 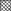 Undetermined species.

Parentage analyses in six stands allowed one or two parents to be identified among the genotyped adult trees in a proportion of the sampled seedlings (Table 3). The success of parent assignment was higher when the number of seedlings analysed was larger, probably due to the positive correlation between the number of genotyped seedlings and the number of genotyped adults. However, according to the profiles of reproductive success (Table 4, Fig. 4), the effective number of parents (1/Σ(*p_i_* ^2^), where *p_i_* is the relative reproductive success of each parent) varied among stands. For instance, seedlings in Great Britain and Sweden were produced by similar numbers of parents, but since more trees in Great Britain produced more seedlings than in Sweden, where reproductive success was more balanced (Table 4), the effective number of parents was higher in Sweden. On average, 64% of the trees in a stand effectively participated in reproduction over the six stands, with high variation among stands, probably due to different sampling schemes. The L-shaped structure of parental reproductive success was a general trend (Fig. 4) that is in line with results obtained from paternity analysis, even if parental reproduction mix paternal and maternal contributions.

**Table 4:** Statistics of the parental reproductive success.

			*Ns*/*Np*				
Site	*Ns*	*Np*	Min-Max	Mean	SD	*Ne*	*Ne*/*Np* %
France	165	98	1–5	1.27	0.71	74.64	76.16
Italy	387	154	1–24	2.83	3.36	63.99	41.55
Spain	65	18	1–6	1.39	1.20	10.59	58.83
Great Britain	191	74	1–33	2.89	4.91	19.27	26.04
Denmark	84	38	1–4	1.21	0.70	28.60	75.26
Sweden	175	75	1–16	1.64	1.97	30.94	41.25
Mean		76.17				43.49	53.18
SD		47.71				24.27	20.31

*Ns*: number of seedlings. *Np*: number of different parents assigned, *Ns*/*Np*, number of seedlings per parent, SD: standard deviation, *Ne*: effective number of parents (1/Σ(*p_i_* ^2^), where *p_i_* is the relative reproductive success of each parent). See Fig. 4: variation of the relative reproductive success of trees.

**Figure 5:**
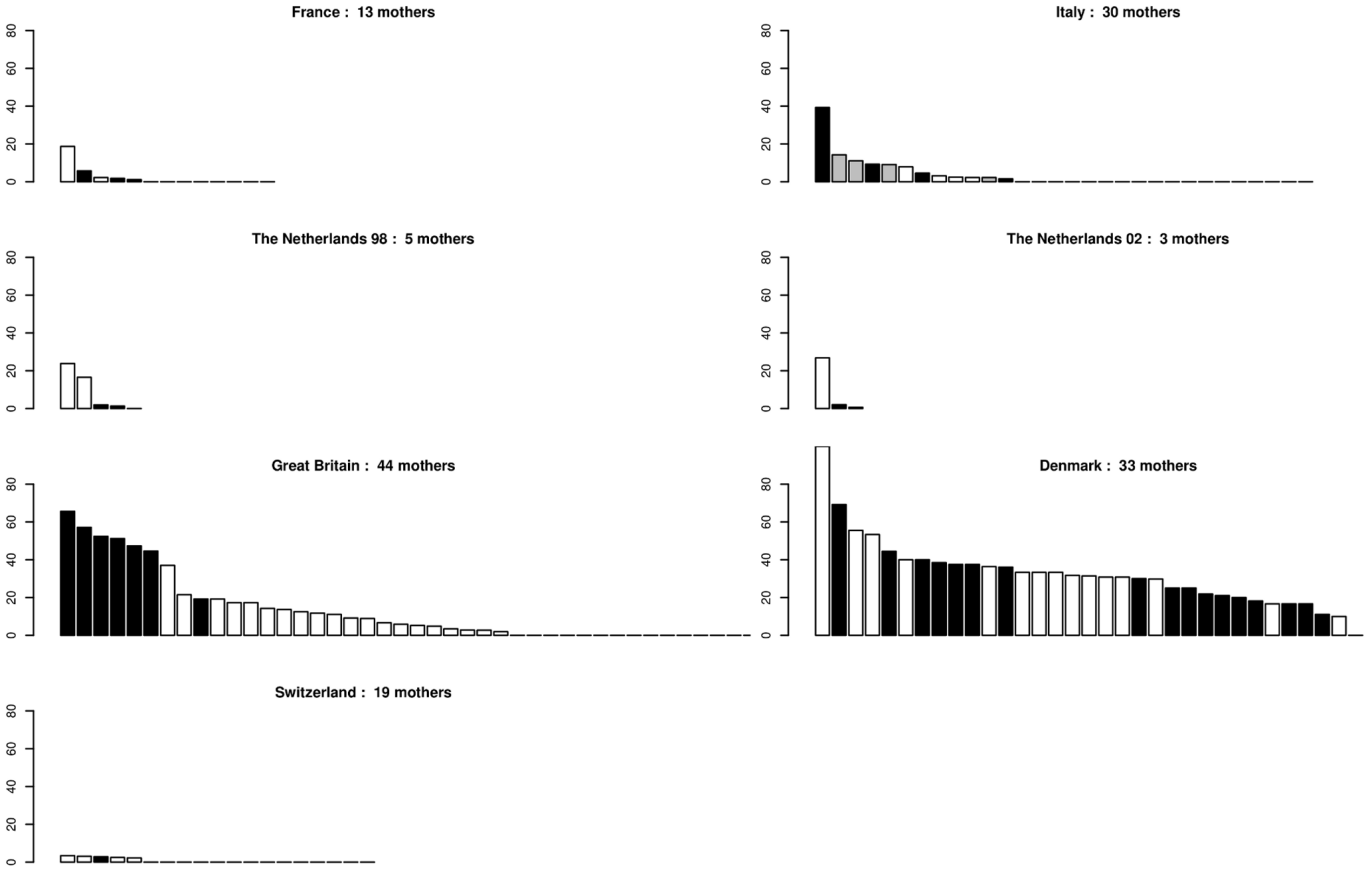
Variation of the percentage of hybrid acorns per mother tree. In each stand, each bar is a mother tree, bar height represents the percentage of acorns collected from the mother tree with a father assigned from a species different from the species of the mother tree (hence a hybrid). □ *Quercus petraea* ▪ *Quercus robur* 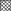 Undetermined species.

**Hybridisation rate.** Hybridisation involving *Q. petraea* and *Q. robur* was observed in the five stands where the two species co-occurred (France, The Netherlands, Great Britain, Denmark, Switzerland; Table 1). In all cases, hybridisation generally involved a *Q. petraea* mother and a *Q. robur* father, but with variable relative frequencies (Table 5, from 1.1 to 12.2 times more *Q. petraea* × *Q. robur* than *Q. robur* × *Q. petraea*). The percentages of hybrid acorns were high in Denmark, The Netherlands (but with substantial interannual difference) and Great Britain. The percentage of hybrid acorns per mother tree also varied largely, i.e. some mothers contributed more to hybridisation than others, and the species identity of these mothers varied among stands (Table 5, Fig. 5). A high number of hybridising mothers was observed in Great Britain, Denmark and to a lesser extent in Italy where hybridisation was between *Q. petraea* and *Q. pubescens*. Strikingly, in each stand a few trees exhibited very high rates of hybridisation. In Great Britain and in Denmark, sampling involved over-representation of acorns from *Q. petraea* mothers and this may account for the high estimation of hybridisation. However, in the Netherlands, this sampling bias was not present, and cannot explain the high levels of hybridisation that were also detected. Heterogeneity of between- and within-stand hybridisation might be explained by species' spatial organisation within the areas, related in particular to the different ecological requirements of the species.

**Table 5:** Cross-fertilization and hybridisation rates.

	Intraspecific		Hybrid (♀ × ♂)			*H*/*M*		
Site	*Qp* × *Qp*	*Qr* × *Qr*	*Qp* × *Qr*	*Qr* × *Qp*	*h*	Mean	SD	*s*
France	47.26	44.21	5.79	1.52	6.94	1.85	4.65	4.57
Italy*	*75.93*	*13.31* *	*2.74* *	*4.11* *	*7.37*	*1.17*	*2.47*	2.54
The Netherlands 98	5.69	63.03	28.91	2.37	33.17	13.20	16.30	3.32
The Netherlands 02	0.65	81.94	14.19	3.22	17.76	9.00	11.36	0.00
Spain	-	100	-	-	-	-	-	4.00
Great Britain	2.68	67.96	21.01	8.35	29.80	4.58	8.76	2.24
Denmark	28.08	30.14	21.69	20.09	41.03	5.55	4.89	2.28
Sweden	-	100	-	-	-	-	-	0.84
Switzerland	67.51	19.24	1.45	0.18	1.04	0.47	0.90	3.04
Mean					19.59	5.12		2.54
SD					15.30	4.63		1.44

Percentages of intraspecific and hybrid crossing: *Qp* (*Q. petraea*), *Qr* (*Q. robur*), hybrid crosses: female ♀ × male ♂. *h*: percentage of hybridisation among acorns with an assigned father. *H*/*M*: mean number of hybridisation events per mother (see Fig. 5: variation of the percentage of hybrid acorns per mother tree), SD: standard deviation. *s*: percentage of selfing.

Qp = Q. petraea, Qr = Q. pubescens.

### Dispersal curve analysis

**Figure 6:**
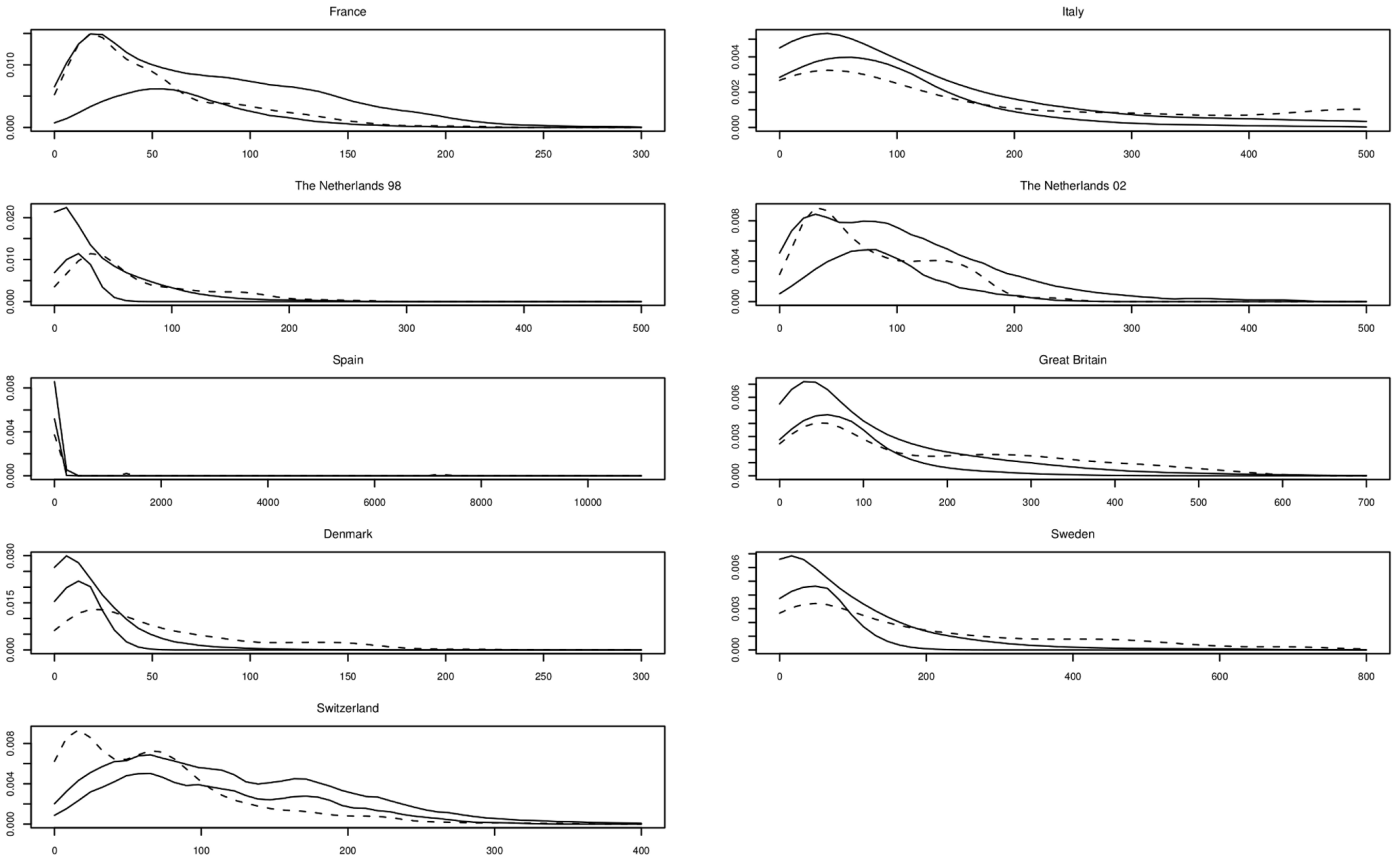
Pollen dispersal distributions. In each stand, observed pollen dispersal is represented by dotted line, confidence intervals are delimited by solid lines. ________ confidence limits ------------- observed distribution.

**Pollen dispersal curves (paternity analysis).** In each stand, the distribution of mother–father distances was fitted to a power exponential function (providing better fit for airborne pollen dispersal than more traditional descriptive dispersal models [33]), and confidence bands were obtained through bootstrapping (Table 6, Fig. 6).

**Table 6:** Pollen and seed dispersal distances.

	Pollen dispersal				Seed dispersal	
Site	Min (m)	Max (m)	*b*	*δ* (m)	*b*	*δ* (m)
France	0.0	216	0.23 (0.07–0.39)	15.6 (7.5–22.4)	7.90 (0.18–39.68)	4.1 (0.7–6.5)
Italy	0.0	836	0.14 (2.52e–06–0.28)	5371.1 (1923.4–9708.2)	0.075 (4.4e–16–0.4)	119.2 (15.1–518.1)
The Netherlands 98	0.0	264	0.14 (0.02–0.27)	671.1 (45.6–5306)	-	-
The Netherlands 02	19.2	233	0.08 (0.007–0.29)	1101.6 (54.2–7018.6)	-	-
Spain	0.0	9977	0.31 (2.56e-20-0.47)	5149.4 (1028.5-9612.0)	-failed-	0.6 (0.5–0.8)
Great Britain	0.0	644	0.01 (3.72e-44-0.09)	1185 (186.7–4722.9)	7.82 (5.6e-15-18.8)	98.6 (88.6–102.2)
Denmark	0.0	224	0.23 (2.21e-14-0.53)	166.3 (61.5–603.7)	11.37 (9.5e-07-26.9)	62.4 (20.1–76.0)
Sweden	0.0	841	0.04 (0.008–0.12)	497.1 (227.2–1969.5)	1.05 (0.3–2.4)	143.1 (66.1–376.1)
Switzerland	0.0	346	0.02 (0.01–0.03)	3674.4 (161.5–9242.7)	-	-

Fit of the observed data to a one-dimensional exponential power distribution with parameters *δ* and *b*.

**Figure 7:**
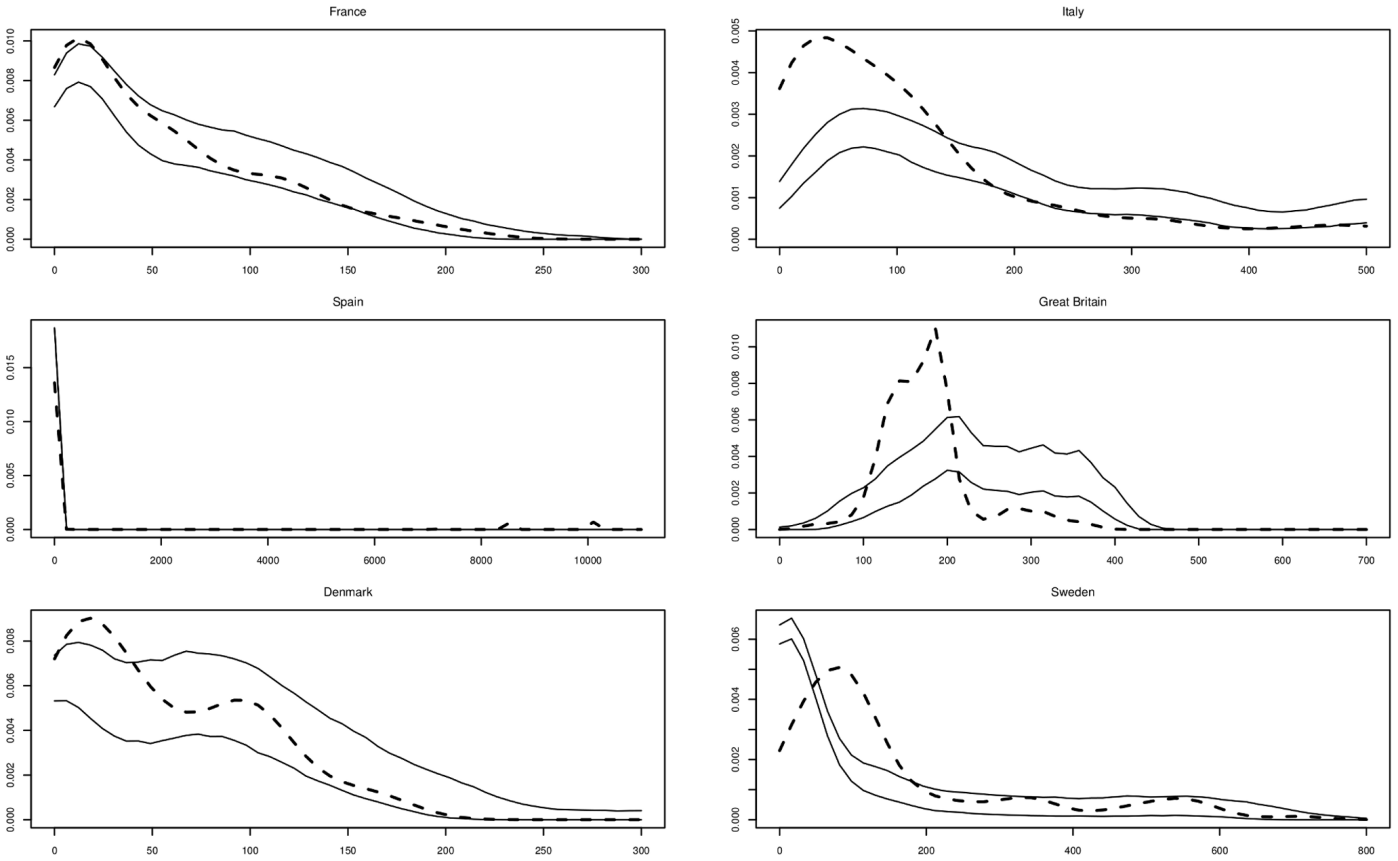
Seed dispersal distributions. In each stand, observed seed dispersal is represented by dotted line, confidence intervals are delimited by solid lines. ________ confidence limits ------------- observed distribution.

Minimum (min) and maximum (max) pollen dispersal distances observed (in meters). *δ*: mean dispersal distance travelled by the pollen*/*seed (in meters), *b*: shape parameter. Mean parameter values and confidence intervals at 95% (numbers within brackets) were obtained after 1000 bootstrap resampling (see Fig. 6: pollen dispersal distributions (observations and confidence intervals) and Fig. 7: seed dispersal distributions (observations and confidence bands)).

The shape parameter *b* estimated for pollen dispersal was always <1, and confidence intervals did not include 1, indicating fat-tailed pollen dispersions. Estimated mean dispersal distances (*δ*) ranged from 16m (France) to almost 5,400 m (Italy). The distances depended on the size of the stands, the two largest stands (Spain and Italy) exhibiting the largest *δ*. In Spain, some long-distance dispersal events (almost 10 km) were observed between the scattered groups of trees, while the remaining observations were local, with no correct fit possible: this stand was therefore not included in the following analysis.

Curves were generally located within the confidence envelopes, with some recurrent exceptions. Short distances were always associated with peaks in the curves, but were less frequent than predicted by the model in five out of nine stands (Italy, The Netherlands 98, Great Britain, Denmark, Sweden) and more frequent in two cases (The Netherlands 02, Switzerland), with France showing no deviation from the model expectations (Fig. 6).

In all stands, mean dispersal distances observed were significantly shorter than those based on a random assignment of fathers to acorns (data not shown), indicating a restricted spatial dispersion of pollen inside stands.

**Seed and pollen dispersal distances (parentage analysis).** Dispersal distances were deduced from single-parent and parent-pair assignments of seedlings, and dispersal curves were fitted to one-dimensional exponential power functions when sufficient numbers of observations were available.

When only a single parent could be assigned to a seedling, the mean parent–seedling distance in all stands was significantly shorter than the distances obtained by random assignment of a parent to the same seedlings (data not shown). The mean distance of seedlings to their single parents was slightly higher for *Q. petraea* than for *Q. robur* parent trees (France, Denmark), but remained of the same order of magnitude. Some very long-distance seed dispersal events were observed in the low-density, highly fragmented Spanish population (>10 km, Fig. 7).

The assignment success was lower with parent pairs than with single parents, thus fewer such events were observed in each stand, providing probably unreliable estimates of reproductive parameters (Table 3). Hybridisation was detected, especially in Great Britain, and selfing was also identified with values higher than those estimated in paternity analyses. Seed and pollen dispersal distances were deduced from parent-pair assignments, assuming that the mother tree was the closer of the two identified parents and that pollen had travelled between the two mating trees. In all stands, mean pollen dispersal distance was significantly shorter than that modelled by random assignment of fathers (data not shown).

The mean ratio of pollen to seed dispersal distances was significantly higher than expected on the basis of random parent assignments in France and Great Britain, where pollen was dispersed 17 and five times further than seed, respectively. However, we acknowledge that this estimate might be biased, as it was based on the assumption that, in all cases, the maternal parent was the closer of the two parents to the seedling; an assumption we consider legitimate.

Moreover, this ratio of pollen to seed dispersal distance was much greater for *Q. petraea* than for *Q. robur* parent pairs in France (25× versus 9×) but only slightly greater in Great Britain (5× versus 4×). However, these values are based on small numbers of observations and should therefore be taken with caution.

When sufficient numbers of observations were available, the distributions of pollen and seed dispersal were fitted with a one-dimensional exponential power function. Estimations of mean distance travelled by pollen and seed (*δ*) and of respective shape parameters (*b*) were deduced and dispersal distributions of each stand were drawn (Table 6, Fig. 6 and 7).

The mean distance between seedling and single parent (which we assumed to be a seed dispersal distance) was significantly smaller than that modelled on a random assignment of parent to seedlings (data not shown).

With the exception of Italy, the shape parameters *b* of seed dispersal were >1, indicating light-tailed dispersions for acorns. In some cases, observed distributions of seed dispersal were not located between the two estimated confidence limits (Fig. 7).

## Discussion

Gene flow plays a major role in many aspects of evolutionary biology and is therefore one of the key components for understanding population processes within and among co-existing species. Here, we present concise analyses of pollen and seed dispersal, individual mating success and hybridisation studied in seven mixed and one pure white oak stands across Europe. Some stands were firmly located within the natural distribution range of the species (Denmark, France, The Netherlands, Switzerland), whereas others were located at the limits of the distribution (Great Britain, Italy, Spain, Sweden). This situation allowed us to draw general conclusions on gene flow in white oaks at the species distribution scale. While our results largely confirm previous studies that were restricted to single stands, they also emphasize the large variation that exists among stands in all parameters, particularly highlighting restricted dispersal within stands despite fat-tailed dispersal curves and large fractions of gene immigration that were mostly based on pollen. In turn, we demonstrate that hybridisation between species is not entirely unidirectional, but is driven by species composition. Although hybridisation is consistently lower than intraspecific gene flow, it is variable among trees and stands. In conclusion, an unavoidable aspect inherent to range-wide studies is the spatial, temporal and environmental heterogeneity among populations. This variation increases statistical noise, which makes interpretation difficult, especially for deriving global trends.

### Pollen and seed flow

On average, 57% of the pollination events detected in paternity analyses, i.e. using seeds from known mother trees, involved individuals from outside the stands (Table 2). This is consistent with previous observations for the same or other oak species ([34], [35], [5], [7], [36] see Table 4 in [37] for a comprehensive comparison, or [9] in Fagaceae (*Quercus*, *Fagus*, *Castanea*)).

More surprisingly, parent assignments in seedlings indicated that on average at least 38% grew from acorns that originated from outside the study plots. The same data gave an average pollen immigration estimated as high as 85% on average, representing a mean total gene immigration of 61%, although with a high variability across stands (SD  =  15). These estimates were deduced from the fact that 38% of seedlings had no parent, 46% had a single parent, which was assumed to be the mother, and only 15% had both parents assigned from within the stand. Such high levels of unassigned maternity suggest that seed dispersers, most likely jays [38], actively move a substantial fraction of acorns among stands as compared to within stands, whereas wind-borne pollen contributes an even greater fraction to the total gene immigration.

### Reproductive success

The reproductive success of individual fathers had an L-shaped distribution in all stands, i.e. a limited number of fathers sired several offspring, whereas the remaining majority of trees that acted as fathers sired only a single or a very restricted number of offspring from the sampled mother trees. The number of fathers that contributed to reproduction was highly variable among stands (from 21 to 286). On average, the effective number of fathers represented less than half the trees in the stands, again with some extreme values. The number of fathers assigned per mother tree showed the same trend in all stands, with a smooth decrease when sorting paternal contributions per mother in descending order. However, some stands contained mothers in which almost all of their acorns had assigned fathers, while lower rates of paternal assignment were observed elsewhere in the stand (Fig. 3). The location of the mother tree within the stand, in particular its position relative to the borders of the stand, or its proximity to other trees of the same species could explain the differences observed among mother trees in the same stand.

As in the paternity analysis, the reproductive success rates of assigned parents in all stands followed L-shaped distributions, with only a few individuals acting as parents of many offspring and the majority of individuals being the parent of only a single or a few offspring (Fig. 4). Globally, this distribution trend resulted in slightly more than half of the assigned parents effectively reproducing, as inferred from parentage analyses of seedlings.

Such uneven parental contributions are commonly observed in paternity, maternity and parentage analyses [39], [40], [41], [42], [43]. It should be noted that this pattern probably reflects to some degree a sampling effect, because genotyping is usually limited to only a small fraction of mother trees, dispersed seeds or seedlings. Nevertheless, uneven contributions to reproductive success are not unrealistic, nor limited to plants [44], and could have a strong influence on effective population size and thereby on response to selection of adaptive traits. However, since we observed that multiple paternity*/*parentage assignments for one tree remained a restricted phenomenon and that trees involved in multiple paternity*/*parentage probably differ across sampling seasons (our two sampling years in the Netherlands diverge for the number of different fathers assigned — 92 and 68 — ; see also [26], [45]), the reduction in effective population sizes over many generations may be limited [46]. Our data are, however, not sufficiently detailed to draw firm comparisons regarding among-year variation in any of the parameters evaluated here, and multi-year studies of mating pattern are still needed to develop a full understanding of its long-term impact on the genetic diversity and adaptability of oak populations.

Mean selfing was 2.5% according to paternity analysis (Table 5), but could not be properly estimated by parentage analysis because the number of seedlings with two parents assigned was generally too small (Table 3). Our dispersal model gave very low estimates of selfing (Table 6), an outcome that was expected for these oak species, which are known to be highly outcrossing [4], [8]. These low numbers of selfed individuals at the seed stage are expected to decrease even further during establishment as a consequence of inbreeding depression (e.g. [47]). We therefore confirm the minor role that selfing plays in the mating system of oaks.

### Dispersal curves

As in [9], our study of contemporary gene flow in oak stands from different parts of Europe revealed considerable variability in dispersal curve parameters. Nevertheless, some trends were remarkably consistent. In all stands, pollen dispersal was fat-tailed, while seeds were dispersed according to a light-tailed distribution, with shorter mean dispersal distances. Accordingly, even though acorn immigration was high, dispersal modelled inside the stands attributed less importance to long-distance dispersal events for seed than for pollen, as expected for a species with wind-dispersed pollen and mostly barochorous acorns. However, this outcome might be partly due to the bias introduced in the parentage analyses, i.e. the assumption that the closer of two parents assigned to a seedling is the mother tree. In addition, long-distance dispersal was incompletely assessed due to the inherent limitation imposed by the fact that the maximum distance between pairs of trees within the study plots was the greatest dispersal distance that could be detected. Direct estimates of seed dispersal can be achieved by genotyping tissues of maternal origin, e.g. the cotyledons remaining on germinated seedlings [48], [49], which was not part of our experiment. Alternatively, models can be used to distinguish male and female contributions [50], but in such an approach, the outcome depends also on how well model assumptions are met.

Long-distance dispersal of acorns as observed in the Spanish stand is probably more common in oaks than inferred from dispersal curves, despite the large size of seeds [51], [52]. However, mean pollen and acorn dispersal distances observed inside the stands were always shorter than expected by randomly selecting fathers for acorns or parents for seedlings, as has been previously observed for *Quercus salicina* pollen [35]. Maternal trees received the majority of their pollen from a small number of local donors, the rest being contributed by many individuals located further away, a situation regularly observed in previous studies [4], [53], [35], [54]. Similarly, acorns producing seedlings exhibit shorter dispersal distances within stands than is expected from models based on random assignment of mothers.

In Italy, Great Britain, Denmark and Sweden, observed pollen dispersal curves were located outside and below the confidence limits (Fig. 6). This means that the dispersal model we used is unsuitable in these cases. These stands had a particular sampling strategy for seedlings (Supporting Information, Fig. S2), possibly creating a bias in the parentage analysis results used for pollen dispersal curve estimation. Additionally, there was only a small number of mother trees in The Netherlands (five in 1998 and three in 2002), and these were located on the edge of the local study area (Supporting Information, Fig. S1). Such particular locations can represent instances in which dispersal hypotheses are violated (isolated places compared to central ones, with different wind behaviour, for instance) causing deviations between simulated and observed dispersal kernels.

Discrepancies between observed seed dispersal distribution and confidence limits are particularly conspicuous in Italy and in Great Britain (Fig. 7). In these two stands, seedlings were sampled in restricted areas (Supporting information, Fig. S2), giving only a very local insight into seed dispersal, which contradicts the assumptions used in the model. In Denmark, part of the analysed seedlings were sampled in a single patch whilst the remainder were obtained from scattered locations within the stand, probably giving rise to a better fit of dispersal curve within the confidence limits. In Sweden, the fit is surprisingly inadequate despite a regular sampling scheme. The curve suggests a systematic effect that would increase seed dispersal compared to the predicted values. This could involve dispersal of seeds via transportation in the Dalälven River along which the stand is located. Our model was built assuming independence between dispersal events, a hypothesis which probably does not hold given the results found in the Swedish stand: it seems likely that sampling scheme has a notable impact on the outcomes of dispersal studies [55], [8].

### Hybridisation

When *Q. petraea* and *Q. robur* co-occurred, hybridisation events could be detected in both acorns and seedlings, which confirms that hybrid acorns can germinate and survive at least to the seedling stage. In all stands, hybridisation was most common when it involved *Q. petraea* mothers being fertilized by pollen from *Q. robur* (Fig. 5). This outcome contradicts previous findings that *Q. petraea* shows higher siring success compared to *Q. robur* in hybridisation within mixed stands [56], [57]. In Great Britain, the particularly high incidence of hybridisation in *Q. petraea* mothers could be due to the highly imbalanced species composition of the stand (93% *Q. robur* trees), largely biasing pollen availability [10], and to the sampling of a larger than average proportion of acorns from *Q. petraea* trees. We observed substantial variation in hybridisation rates per mother among stands (Table 5, Fig. 5). It may be that some mother trees are themselves hybrids (France [58]; Switzerland [18]), substantiating the relevance of correct prior taxon assignment of individuals. Three stands (The Netherlands 98, Great Britain and Denmark) exhibited a global percentage of hybrid acorns of ≥ 30%. Widespread evidence of hybridisation exists in oak species, highly variable in direction and intensities (for recent examples see [59], [5], [7], [10], [6], [60]). Nevertheless, local hybridisation rates are certainly affected by a variety of factors such as tree density, flowering time, micro-environmental selection, species assignment, sampling strategy and the relative spatial arrangement of the species.

While all mixed stands showed some degree of hybridisation, the rates varied greatly among stands. The highest values occurred in the two northernmost stands (Great Britain and Denmark), which could be related to their location at the distribution limit of the species: northern ranges were more recently colonized and hence could be characterized by higher rates of hybridisation [61].

An important source of variation was observed between years in the replicate available (The Netherlands 98 and 02), where the rate differed by a factor of two. However, our study design is not suited for testing the relevance of such spatial and temporal effects.

In the seven stands containing mixed species, the relative frequency of *Q. petraea* exhibited a negative, though not significant, correlation with the frequency of either *Q. robur* × *Q. petraea* or *Q. petraea* × *Q. robur* hybrids (Table 5). This trend suggests (but does not prove) the importance of the relative frequencies and spatial organisation of tree species on pollen composition and hence on hybridisation, as shown for instance by [10].

## Conclusions

Our data show a general trend in effective dispersal of wind-borne white oak pollen within stands. Pollen dispersal curves largely confirm the predominantly fat-tailed dispersal curves exhibited by wind-pollinated tree species [62], which do not substantially deviate from patterns observed in insect-pollinated species [63]. However, as the data for total gene immigration and those from the large-scale analysis in the Spanish stand indicate, pollen originating from within the stand only accounts for a fraction of the effective pollen immigration. Seed immigration was also high according to parentage analysis in several stands — 38% of acorn immigration — and this is substantially higher than might be expected for a species with such large seeds, even more so when considering the below-expectation distances of seed dispersal within stands. Accordingly, processes other than dispersal due to gravity (barochory) must play an important role in oaks. The dispersal activities of jays [64], [38], [49] and dispersal in the water flow of adjacent rivers might account for the longer-distance (several km) dispersal events detected.

Confidence limits as introduced in our study are rarely presented for dispersal kernels. Since our observed dispersal curves were located outside confidence limits in several cases, the one-dimensional exponential power function classically used to fit these curves might be sensitive to deviations from model assumptions. Similarly, [65] underline high uncertainty in kernel estimation using simulation envelopes. Consequently, even if this kind of approach gives correct estimates of the shape of curves [62] – fat-tailed dispersal for wind-borne oak pollen and light-tailed dispersal for mostly barochorous acorns –, our results suggest that the quality of global dispersal curve modelling requires improvement.

Total gene immigration, combining pollen and seed immigration in parentage analyses, was of the same order of magnitude as pollen immigration estimated in the paternity analysis. However, parentage analysis of seedlings gave much higher estimates of pollen immigration than did the paternity analysis of acorns (85% compared to 57%). Barring methodological artefacts, we anticipate that processes during the development from seed to seedling may increase apparent gene flow by favouring survival of immigrants. One hypothesis is that pathogens or herbivores associated with mother trees are more adapted to seedlings that are related to the mother tree than to immigrants, thereby favouring the survival of the latter (Janzen–Connell hypothesis; [66], [67]). Here, experimental trials may help identify respective agents or selective pressures that act on seeds and seedlings from different sources.

Variation in sampling strategies and stand geometry may have influenced the homogeneity and heterogeneity of our parameter estimates. However, no significant correlations (corrected for multiple testing) between any stand characteristics and any gene flow parameters were detected. Biological and environmental factors may also have influenced our estimates. For instance, jay populations have increased by around 20% between 1980 and 2000 in Europe (European Bird Census Council (EBCC) http://www.ebcc.info/index.php?ID=91). The active involvement of these birds in acorn movement probably increased seed gene flow during this period. We also observed a strongly skewed contribution of fathers to the next generation, which might fluctuate from year to year. Genetic compatibility and phenological synchronization may account to some degree for the observed bias in paternity among trees, and such aspects may add a stochastic component to the overall variation observed in our analyses. Here, dedicated research to account for such effects is required in the future.

In conclusion, the present study highlights the high level of gene flow within and among stands of white oaks, including locally high rates of hybridisation. Consequently, forest services need to be aware that delimiting stands as reserves for the conservation of genetic resources or for seed collections inevitably incorporates a large degree of uncertainty regarding the effect of pollen gene flow on the local genetic composition of seed lots. Dynamic conservation of forest tree genetic diversity needs management rules deduced from results of studies like the one we conducted [68]. From our study, we also conclude that optimal sampling strategy for solid comparisons are important but difficult to achieve when working with natural forest ecosystems [69]. In turn, such a delicate situation allows us to formulate new hypotheses and to develop particular sampling designs, e.g. to test the specific effects of species mixture (spatial composition, ratio) or of study area (size, shape) on gene flow within and among stands and species. The type of study conducted in the present paper is intrinsically bound to heterogeneous environmental conditions as well as to the necessary underlying assumptions of the data analysis. Therefore inferences regarding general trends should be made cautiously. Recent technological advances allowing many more loci to be genotyped at low cost, in particular genome-wide single nucleotide polymorphisms (SNPs), will further improve statistical inference. We therefore see great prospects for further study of gene flow to improve our understanding of this process, which is important also in view of global change, through its key role in species range expansion and in local adaptation.

## Supporting Information

Figure S1 **Maps of the 9 paternity analysis experiments (sizes in meters).+** : adult trees **×** : mother trees sampled.
(TIF)

Figure S2 **Maps of the 6 parentage analyses experiments (sizes in meters). +** : adult trees **×** : seedlings sampled.
(TIF)

File S1 **Tables S1-S5.** Table S1, Stands description: main use of the corresponding forest, silvicutural treatments, existence of regeneration, history and protection status. Table S2, Stand details for paternity and parentage analyses. Table S3, Marker polymorphisms for each stand. Table S4, Results of test simulation (highest values) and corresponding type of test used to assign fathers in each stand. Table S5, Results of test simulations (highest values) and the corresponding type of test used to assign parentage in each stand.
(DOC)
