# Correction: High Rates of Gene Flow by Pollen and Seed in Oak Populations across Europe

**DOI:** 10.1371/annotation/2f561950-468c-4ef8-9a91-3535e8c51ece

**Published:** 2014-01-22

**Authors:** Sophie Gerber, Joël Chadœuf, Felix Gugerli, Martin Lascoux, Joukje Buiteveld, Joan Cottrell, Aikaterini Dounavi, Silvia Fineschi, Laura L. Forrest, Johan Fogelqvist, Pablo G. Goicoechea, Jan Svejgaard Jensen, Daniela Salvini, Giovanni G. Vendramin, Antoine Kremer

A file was erroneously omitted from the list of Supporting Information. The description is 'Datafiles for "High rates of gene flow by pollen and seed in oak populations across Europe" paper' and can be viewed here: http://plosone.org/corrections/pone.0085130.s004.cn.zip

