# Correction: High Rates of Gene Flow by Pollen and Seed in Oak Populations across Europe

**DOI:** 10.1371/journal.pone.0091301

**Published:** 2014-02-28

**Authors:** The PLOS ONE Staff

Reference 69 is incorrect. The correct reference is: Leonarduzzi C, Leonardi S, Menozzi P, Piotti A (2012) Towards an optimal sampling effort for paternity analysis in forest trees: what do the raw numbers tell us? iForest, 5: 18-25. DOI: 10.3832/ifor0606-009
