# Supplementary figures and images for: High Rates of Gene Flow by Pollen and Seed in Oak Populations across Europe
Source: PLoS One. 2014 Jan 13;9(1):e85130. doi: 10.1371/journal.pone.0085130 (PMC3890301; doi:10.1371/journal.pone.0085130)

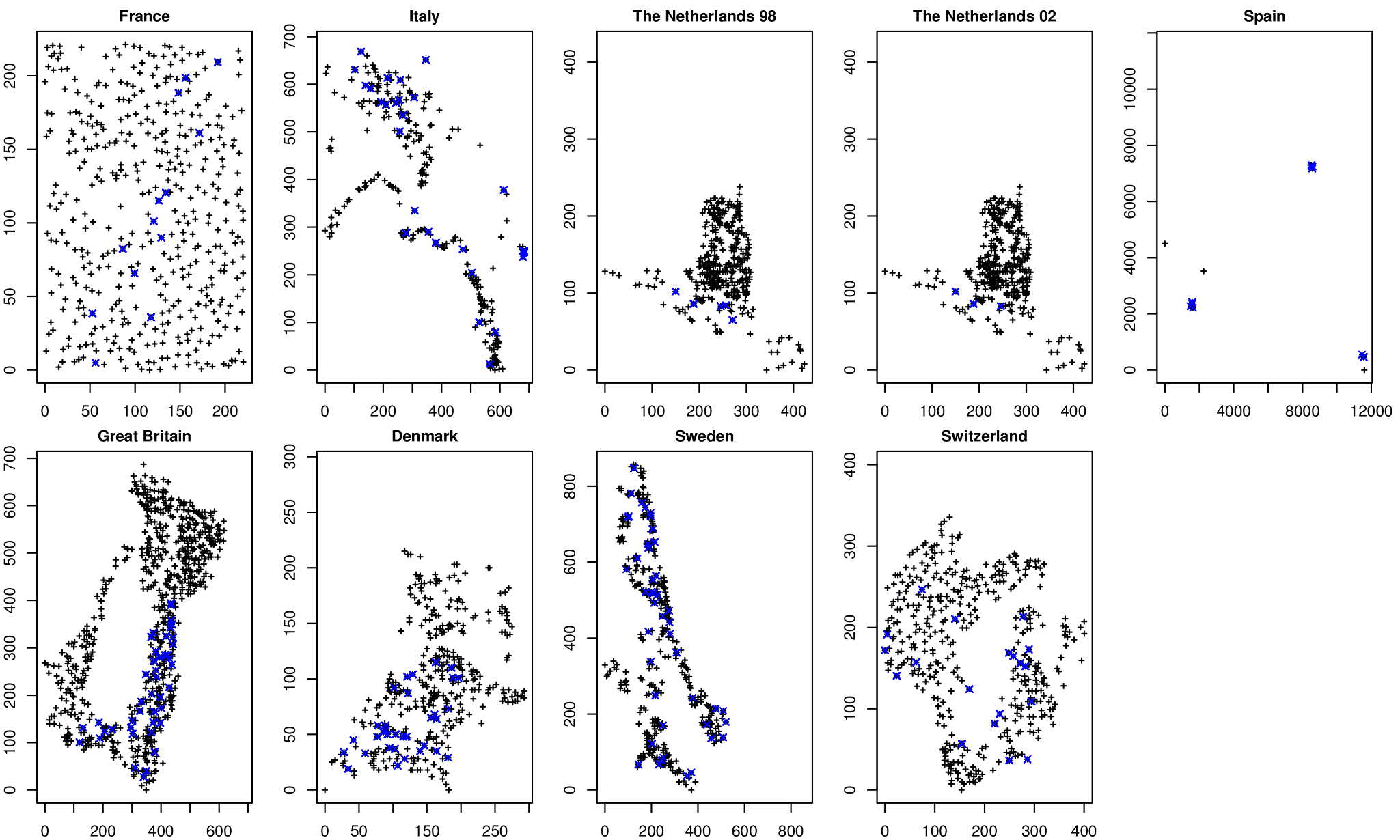

Supplement: Figure S1 — Maps of the 9 paternity analysis experiments (sizes in meters).+ : adult trees × : mother trees sampled. (TIF) [file pone.0085130.s001.tif]

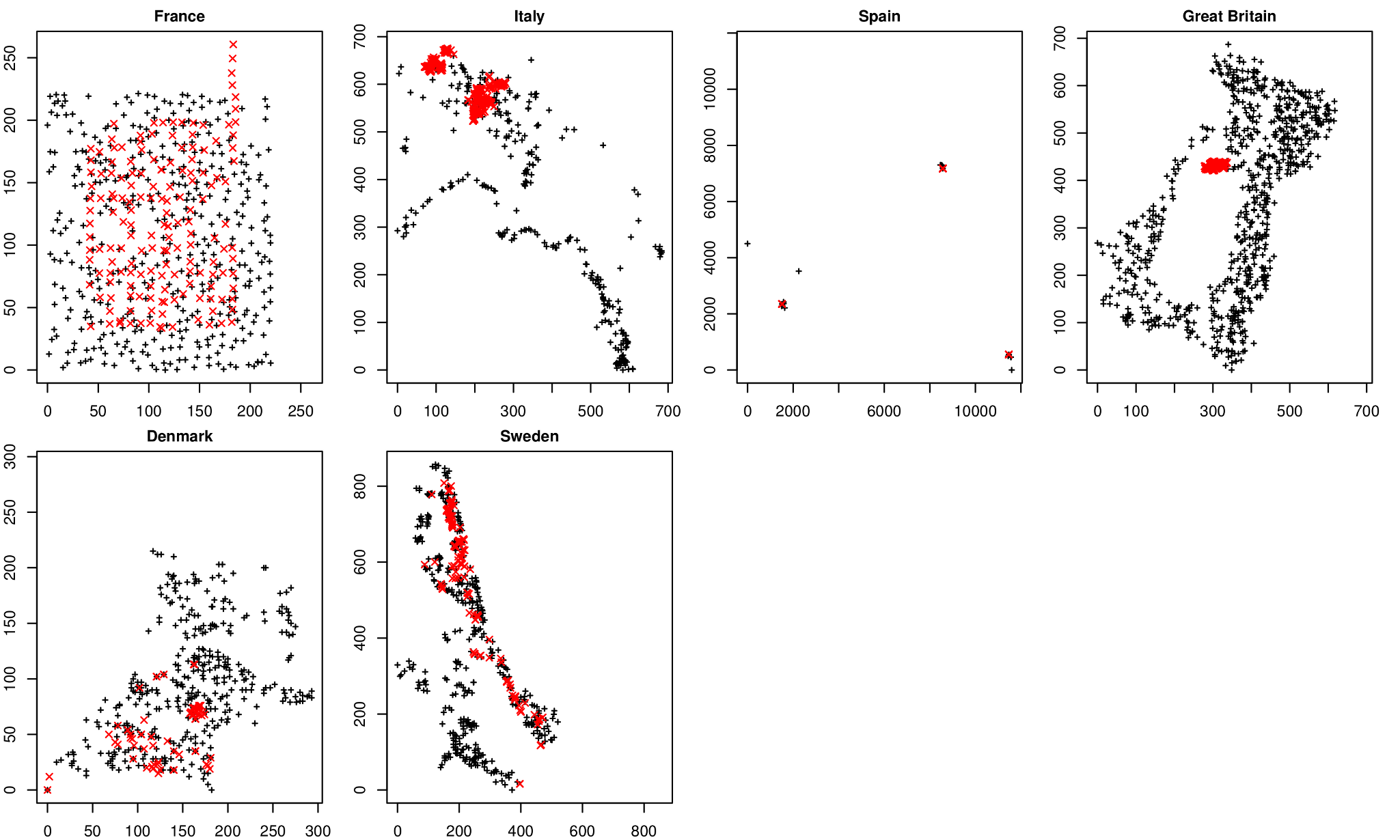

Supplement: Figure S2 — Maps of the 6 parentage analyses experiments (sizes in meters). + : adult trees × : seedlings sampled. (TIF) [file pone.0085130.s002.tif]
