# Supplementary material for: High Rates of Gene Flow by Pollen and Seed in Oak Populations across Europe
Source: PLoS One. 2014 Jan 13;9(1):e85130. doi: 10.1371/journal.pone.0085130 (PMC3890301; doi:10.1371/journal.pone.0085130)
Supplement: File S1 — Tables S1-S5. Table S1, Stands description: main use of the corresponding forest, silvicutural treatments, existence of regeneration, history and protection status. Table S2, Stand details for paternity and parentage analyses. Table S3, Marker polymorphisms for each stand. Table S4, Results of test simulation (highest values) and corresponding type of test used to assign fathers in each stand. Table S5, Results of test simulations (highest values) and the corresponding type of test used to assign parentage in each stand. (DOC) [file pone.0085130.s003.doc]

**SUPPORTING INFORMATION**

**Table S1** Stand description: main use of the corresponding forest, silvicutural practise, existence of regeneration, history and protection status.

| **Country** | **Main use** | **Recent silvicultural practise** | **Regeneration** | **History** | **Protection status** |
| --- | --- | --- | --- | --- | --- |
| France | Production, recreation | Natural regeneration, several cuttings | Natural | Old coppice with standard forest | None |
| Italy | Protection, recreation | None | Natural | Wood and coal production since 19^th^ century | Protected (natural forest area) |
| The Netherlands | Protection, conservation | None | Rare natural regeneration, no silvicultural treatment | Old coppice with standard forest, afforestation | Strict forest reserve, no management |
| Spain | Environmental (biodiversity) | None | Natural | Patches remaining from ancient large forests (>100 years) | None |
| Great Britain | Recreation, conservation | None | Some regeneration in small patches | Ancient deer park with records of existence since 1512 | Site of Special Scientific Interest (SSSI) |
| Denmark | High forest | None | Single gap with seedling | Old forest | Natural indigenous forest |
| Sweden | Protection, recreation | Selective thinning of conifers | Natural | Abandoned pasture, coniferous trees beginning to dominate | Environmental (biodiversity) |
| Switzerland | Production, recreation | Thinning | Natural, but rare | Old-growth forest | None |

**Table S2** Demographic statistics of the stands. *Ngp*: Number of genotyped parents. *Nm*/*Nom*: Number of sampled mothers/mean number of offspring per mother. *No*: Total number of offspring analysed; *Ns*: Number of genotyped seedlings. See Fig. S1.

|  |  | Paternity | | Parentage | |
| --- | --- | --- | --- | --- | --- |
| Site | *Ngp* | *Nm*/*Nom* | *No* | *Ns* | Sampling strategy |
| France | 354 | 13/76 | 992 | 165 | grid |
| Italy | 295 | 30/28 | 841 | 387 | 2 patches |
| The Netherlands 98 | 369 | 5/41 | 700 | - | - |
| The Netherlands 02 | 369 | 3/147 | 440 | - | - |
| Spain | 239 | 11/24 | 264 | 65 | 3 patches |
| Great Britain | 754 | 44/26 | 1136 | 191 | 1 patch |
| Denmark | 365 | 33/17 | 553 | 84 | regular |
| Sweden | 631 | 47/23 | 1061 | 175 | regular |
| Switzerland | 434 | 19/81 | 1543 | - | - |

**Table S3** Exclusion and identity probabilities. *UIP*: unbiased identity probability. *OIP*: Observed identity probability.

| Site | Nb of loci | Exclusion probabilities | | |  |  |
| --- | --- | --- | --- | --- | --- | --- |
|  |  | Single parent | Paternity | Parent pair | *UIP* | *OIP* |
| France | 6^1^ | 0.998243 | 0.999910 | 1.000000 | 0.00000 | 0.00002 |
| Italy | 5^2^ | 0.987387 | 0.998713 | 0.999989 | 0.00000 | 0.00002 |
| The Netherlands | 5^3^ | 0.975451 | 0.997031 | 0.999960 | 0.00000 | 0.00010 |
| Spain | 6^4^ | 0.997946 | 0.999893 | 1.000000 | 0.00000 | 0.00000 |
| Great Britain | 5^5^ | 0.993198 | 0.999390 | 0.999997 | 0.00000 | 0.00009 |
| Denmark | 8^6^ | 0.997005 | 0.999892 | 1.000000 | 0.00000 | 0.00000 |
| Sweden | 6^7^ | 0.993038 | 0.999507 | 0.999998 | 0.00000 | 0.00015 |
| Switzerland | 5^8^ | 0.991458 | 0.999099 | 0.999996 | 0.00000 | 0.00011 |

^1^MSQ4, MSQ13, ssrQpZAG104, ssrQpZAG9, ssrQpZAG1/5, ssrQpZAG36

^2^MSQ13, ssrQpZAG104, ssrQpZAG9, ssrQpZAG1/5, ssrQrZAG96

^3^MSQ13, ssrQpZAG104, ssrQpZAG9, ssrQpZAG1/5, ssrQrZAG96

^4^MSQ4, MSQ13, ssrQpZAG104, ssrQpZAG9, ssrQpZAG1/5, ssrQpZAG36

^5^MSQ4, MSQ13, ssrQpZAG104, ssrQpZAG9, ssrQpZAG36

^6^MSQ4, MSQ13, ssrQpZAG104, ssrQpZAG9, ssrQrZAG11, ssrQrZAG30, ssrQrZAG39, ssrQrZAG96

^7^MSQ4, MSQ13, ssrQpZAG104, ssrQpZAG9, ssrQpZAG1/5, ssrQrZAG20

^8^MSQ13, ssrQpZAG104, ssrQpZAG9, ssrQrZAG30, ssrQrZAG96

Dow *et al.*, 1995: MSQ4, MSQ13,
Steinkellner *et al.* 1997: ssrQpZAG104, ssrQpZAG9, ssrQpZAG1/5, ssrQpZAG36

Kampfer *et al.* 1998: ssrQrZAG20, ssrQrZAG11, ssrQrZAG30, ssrQrZAG39, ssrQrZAG96

**Table S4** Simulation results for paternity analysis.

| Site | Test | *Sim* | *Cfc* | *Pca* | *Cgf* (*β*) | *α* |
| --- | --- | --- | --- | --- | --- | --- |
| France | *l* & *d* | 97.88 | 98.84 | 98.87 | 0.07 | 0.41 |
| Italy | *d* | 71.43 | 74.88 | 63.05 | 14.76 | 2.12 |
| The Netherlands 98 | *l* & *d* | 88.46 | 90.24 | 88.38 | 3.46 | 2.41 |
| The Netherlands 02 | *l* & *d* | 88.52 | 90.36 | 89.88 | 2.46 | 3.54 |
| Spain | *d* | 98.50 | 99.43 | 98.87 | 0.31 | 0.00 |
| Great Britain | *l* & *d* | 82.52 | 81.38 | 73.99 | 8.22 | 4.82 |
| Denmark | *d* | 98.72 | 99.11 | 98.40 | 0.04 | 0.14 |
| Sweden | *l* & *d* | 64.97 | 75.16 | 81.66 | 4.35 | 10.62 |
| Switzerland | *l* & *d* | 79.45 | 85.12 | 85.81 | 1.28 | 9.79 |
| Mean |  |  | 88.28 | 86.55 |  |  |
| SD |  |  | 9.81 | 12.21 |  |  |

Test: fathers assigned according to their delta scores (*d*) or to both their lod-scores and delta-scores (*l* & *d*), with no mistyping. *Sim* (%): the most likely father is the true father when simulating offspring (100,000) with an identified genotyped father. *Cfc* (%): correct father choice rate (father correctly assigned either inside or outside the studied stand). *Pca* (%): paternity correctly assigned among the assigned paternities. *Cgf* (%): cryptic gene flow or *β* error, i.e. assign a genotyped father when the true father is outside the stand (false assignment). *α* (%): α error, i.e. assign no father when the true father is inside the stand (false rejection). SD: standard deviation.

**Table S5** Results of test simulations (highest values) and the corresponding type of test used to assign parentage of seedlings in each stand. Simulation results for parentage analysis.

| Site | Test | *Simp* | *Simpp* | *Cpc* | *Cppc* | *Cgf* (*β)* | *α* |
| --- | --- | --- | --- | --- | --- | --- | --- |
| France | *l*+*m* | 89.79 | 95.78 | 84.70 | 92.73 | 9.03 | 2.26 |
| Italy | *l* | 54.31 | 52.37 | 53.75 | 69.25 | 0.00 | 31.71 |
| Spain | *l* | 91.03 | 96.97 | 84.62 | 94.62 | 13.14 | 0.68 |
| Great Britain | *l* | 61.31 | 67.92 | 58.77 | 69.63 | 0.00 | 35.31 |
| Denmark | *l* | 93.30 | 97.51 | 89.88 | 96.43 | 9.74 | 0.53 |
| Sweden | *l*+*m* | 49.95 | 37.67 | 60.29 | 63.71 | 4.92 | 19.95 |
| Mean |  |  |  | 72.00 | 81.06 |  |  |
| SD |  |  |  | 16.03 | 15.02 |  |  |

Test: parent(s) assigned according to their lod-scores (*l*) with mistyping (*m*), i.e. (*l*+*m*) or without (*l*). *Simp* (*Simpp*) (%): the most likely single parent (parent pair) is the true parent (parent pair) when simulating offspring (100,000) with identified genotyped parent(s). *Cpc* (*Cppc*) (%): correct parent (parent pair) choice rate (parent (parent pair) correctly assigned either inside or outside the studied stand). Cgf (%): cryptic gene flow or β error, i.e. assign a genotyped parent when the true parent is outside the stand (false assignment). α: α error (%), i.e. assign no parent when the true parent is inside the stand (false rejection). SD: standard deviation.
